# Utilization of Two- and Three-Dimensional Transesophageal Echocardiography in Successfully Guiding Transcatheter Mitral Valve in Bioprosthetic Mitral Valve/Mitral Ring Implantation without Complications in Patients with Thrombus in Left Atrium/Left Atrial Appendage

**DOI:** 10.3390/jcm11237084

**Published:** 2022-11-29

**Authors:** Zeyad M. Elmarzouky, Ming-Chon Hsiung, Amr Darwish, Subash Dulal, Bhanu Maturi, Wei-Hsian Yin, Yung-Tsai Lee, Tien-Ping Tsao, Jeng Wei, Navin C. Nanda

**Affiliations:** 1 Division of Cardiology, Department of Medicine, University of Alabama at Birmingham, Birmingham, AL 35305, USA; 2 Division of Cardiology, Cheng Hsin General Hospital, Taipei 112, Taiwan; 3 UAB Montgomery Internal Medicine Residency Program, Montgomery, AL 36116, USA; 4 School of Medicine, National Yang Ming University, Taipei 112, Taiwan; 5 School of Medicine, Institute of Microbiology and Immunology, National Yang Ming University, Taipei 112, Taiwan; 6 Faculty of Medicine, National Defense Medical Center, Taipei 114, Taiwan

**Keywords:** echocardiography, two-dimensional echocardiography, three-dimensional echocardiography, transesophageal echocardiography, left atrial thrombus, left atrial appendage thrombus, mitral valve in valve, mitral valve in prosthetic mitral valve

## Abstract

Background. The aim of this study is to describe, for the first time to our knowledge, the utilization of both two-dimensional (2D) and three-dimensional (3D) transesophageal echocardiography (TEE) in successfully performing transcatheter mitral valve (MV) in bioprosthetic MV/MV annulopasty ring implantation using the apical approach in 12 patients (pts) with co-existing left atrial appendage (LAA) and/or LA (left atrium) body thrombus, which is considered a contraindication for this procedure. Methods and Results. All pts were severely symptomatic with severe bioprosthetic MV stenosis/regurgitation except one with a previous MV annuloplasty ring and severe native MV stenosis. Thrombus in LAA and/or LA body was noted in all by 2D and 3DTEE. All were at high/prohibitive risk for redo operation and all refused surgery. Utilizing both 2D and 3DTEE, especially 3DTEE, guidewires and the prosthesis deployment system could be manipulated under direct vision into the LA avoiding any contact with the thrombus. The procedure was successful in all with amelioration of symptoms and no embolic or other complications over a mean follow-up of 21 months. Conclusion. Our study demonstrates the feasibility of successfully performing this procedure in pts with thrombus in LAA and/or LA body without any complications.

## 1. Introduction

Bioprosthetic valves and atrio-ventricular valve annuloplasty rings are increasingly used because the need for anticoagulants essential for mechanical valves to avoid thrombosis can be obviated. However, these valves are prone to degeneration and significant stenosis and/or regurgitation may occur within a decade of implantation [1]. Annuloplasty rings have also failed resulting in significant valvular or para-ring regurgitation. Repeat surgery in these patients who are often elderly and have other comorbidities such as chronic renal failure carries a high mortality [2]. Because of this, procedures requiring minimal, or no surgery have come into vogue. One such approach is transcatheter prosthetic mitral valve (MV) implantation in previously surgically implanted degenerated bioprosthetic mitral valves or rings using the transapical or transseptal approach [3,4]. These approaches have proven successful in the short term and in some studies over the long-term [5,6]. It is well known that the presence of intracardiac thrombus is a contraindication to these transcatheter procedures because of fear of thrombus fragmentation and dislodgement resulting in systemic embolization. This represents a challenge for severely symptomatic patients with degenerated mitral prosthetic valves or rings and thrombus in the left atrial appendage (LAA)/left atrium (LA) body who are at high or prohibitive risk for redo MV surgery or refuse surgery. In the present study, we present our experience in these patients in utilizing two-dimensional (2D) and three-dimensional (3D) transesophageal echocardiography (TEE) to carefully bypass the thrombus without disrupting it in any way during transcatheter MV in bioprosthetic MV/mitral ring implantation using the apical approach. This procedure carefully performed under the guidance of 2D and 3DTEE as well as fluoroscopy resulted in no embolic or other complications during the procedure or in the follow up period.

## 2. Materials and Methods (Figure 1, Figure 2, Figure 3, Figure 4 and Figure 5, Videos S1 and S2)

In Cheng Hsin General Hospital, Taipei, Taiwan, between 2016 and 2021, a total of 80 adult patients have undergone transcatheter MV in surgically implanted bioprosthetic MV with degeneration resulting in severe stenosis or regurgitation (77 patients) or in severely stenotic native MV with a surgically inserted mitral annuloplasty ring for severe MV regurgitation (3 patients) using the apical approach. Of these, 68 patients had no thrombus in the LAA or LA body. The remaining group of 12 patients (8 females, 4 males, mean age 65.6 years, range 55 to 78 years) with thrombus in LAA, LA body or both form the basis of our study. All were severely symptomatic (New York Heart Association, NYHA, functional class III or IV) with severe bioprosthesis MV stenosis in 9 and severe mitral regurgitation in 2 patients by two-dimensional transthoracic echocardiography (2DTTE). The remaining patient had severe native mitral valve stenosis status post a surgically inserted Sorin (Sorin Group USA, Inc., Arvada, CO, USA) mitral annuloplasty ring for severe MV regurgitation 4 years previously. One of these 12 patients had a second transcatheter MV in bioprosthetic MV procedure following re-development of severe bioprosthetic MV stenosis and thrombus in LAA 2 years and 8 months after the first procedure. Thus, these 12 patients underwent 13 transcatheter MV in valve procedures. All patients had serious comorbidities which presented a high or prohibitive risk for redo surgery and most importantly all absolutely refused further surgery (Table 1) [7]. The decision to go ahead with transcatheter MV implantation in these circumstances was made by a multidisciplinary team consisting of cardiologists, anesthesiologists and cardiac surgeons. Informed consent for the procedure was obtained from all patients. A total of three other patients with thrombus in LAA/LA body agreed to and underwent successful redo surgery.

**Figure 1:**
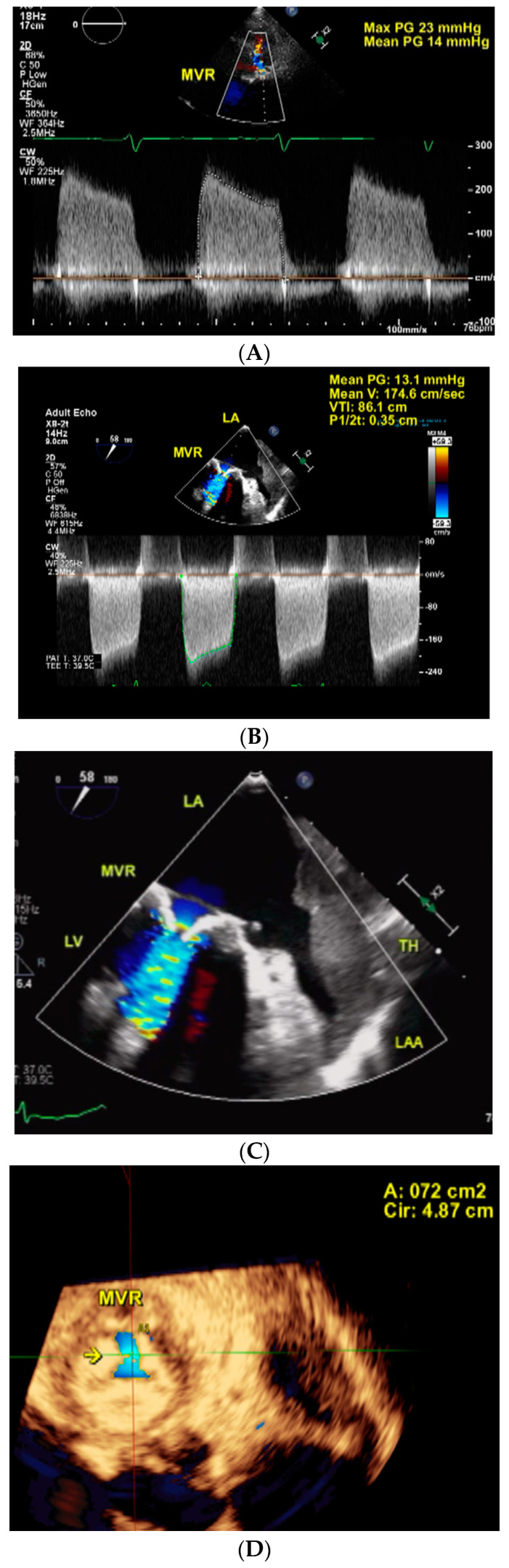

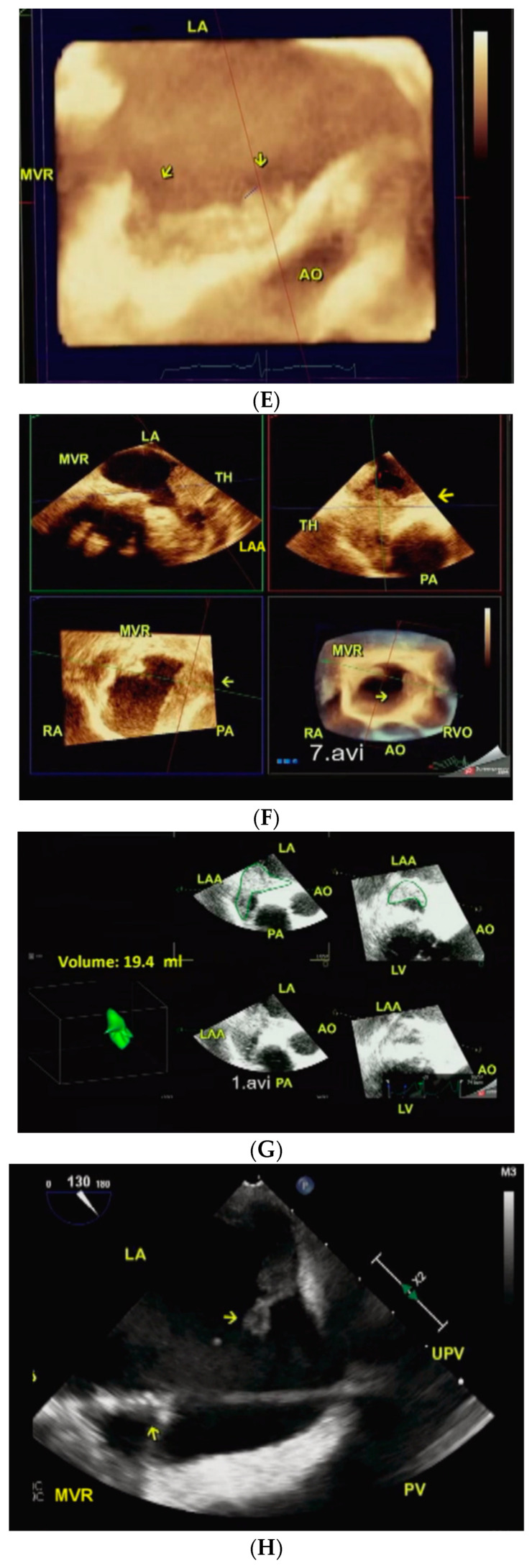

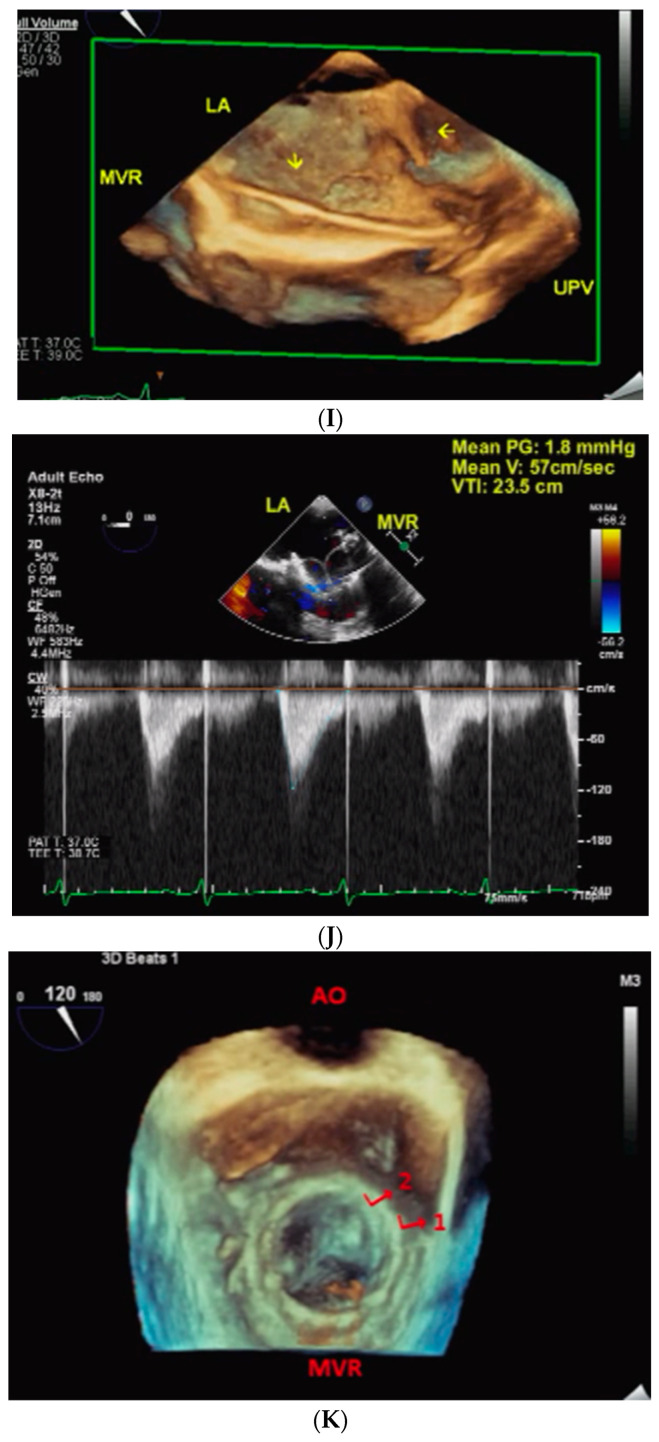
(**A**–**K**), Video S1. 78-year-old female with severe bioprosthetic mitral valve stenosis (Case 10). Pre-procedure. Two-dimensional transthoracic echocardiography with color Doppler guided continuous wave Doppler in (**A**) shows a mean pressure gradient (PG) of 14 mmHg across mitral valve replacement (MVR) consistent with severe stenosis. Two-dimensional transesophageal echocardiography (**B**,**C**) using the same technique as in (**A**) shows a mean pressure gradient of 13.1 mmHg across MVR also consistent with severe stenosis in (**B**). Turbulent flow across MVR as well as a thrombus (TH) in the left atrial appendage (LAA) and adjacent left atrium (LA) body are demonstrated in (**C**). Live/real-time three-dimensional transesophageal echocardiography (**D**–**G**, Video S1). The arrow in (**D**) points to the MVR orifice which measures 0.72 cm^2^ in multiplanar reconstruction (MPR) mode consistent with severe stenosis. Viewing the LA body en face by cropping the full volume dataset from below shows the TH (arrows) extending to near the MVR in (**E**). Video S1 shows both fixed (left arrow) and mobile (right arrow) components of the TH. The right upper panel in F shows the TH involving LAA almost completely and extending to the adjacent LA body (arrow). The arrow in the right lower panel shows the TH adjacent to the aorta (AO). The arrow in the left lower panel shows the TH adjacent to PA. The combined volume of the TH in LAA and adjoining LA body measured 19.4 mL as shown in (**G**). During Procedure. Two-dimensional transesophageal echocardiography (**H**). The lower arrow in H shows the catheter deployment device passing through MVR and lodged in the left upper pulmonary vein (UPV). The upper arrow points to the TH which was bypassed during the procedure. Live/real-time three-dimensional transesophageal echocardiography (**I**). The vertical arrow in (**I**) points to the catheter deployment device bypassing and avoiding the TH (horizontal arrow). Post-procedure. Two-dimensional transesophageal echocardiography (**J**). Color Doppler directed continuous wave Doppler examination in 4 chamber view in J shows a marked decrease in the mean pressure gradient across the new MVR to 1.8 mmHg indicative of relief of stenosis. Live/real-time three-dimensional transesophageal echocardiography (**K**). In (**K**), #1 and #2 represent the old and new prosthetic valve rings, respectively, in full volume short-axis view. LV = left ventricle; PA = pulmonary artery; RA = right atrium; RV = right ventricle; RVO = right ventricular outflow. PV = pulmonary valve.

**Figure 2:**
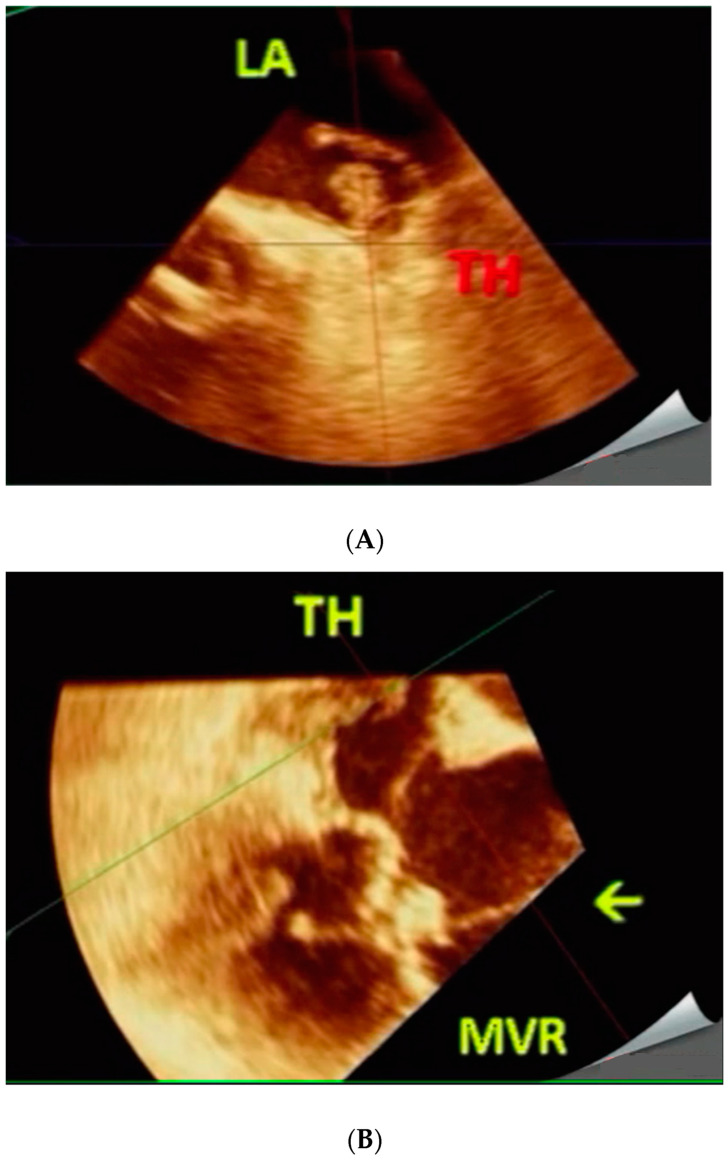
(**A**,**B**). 56-year-old female with severe MVR stenosis (Case 2). Pre-procedure. Live/real-time three-dimensional transesophageal echocardiography (**A**,**B**). A TH is noted in LAA and adjoining LA body in A. During procedure. The arrow in B points to the catheter deployment device passing through MVR avoiding the TH. Abbreviations as in Figure 1.

**Figure 3:**
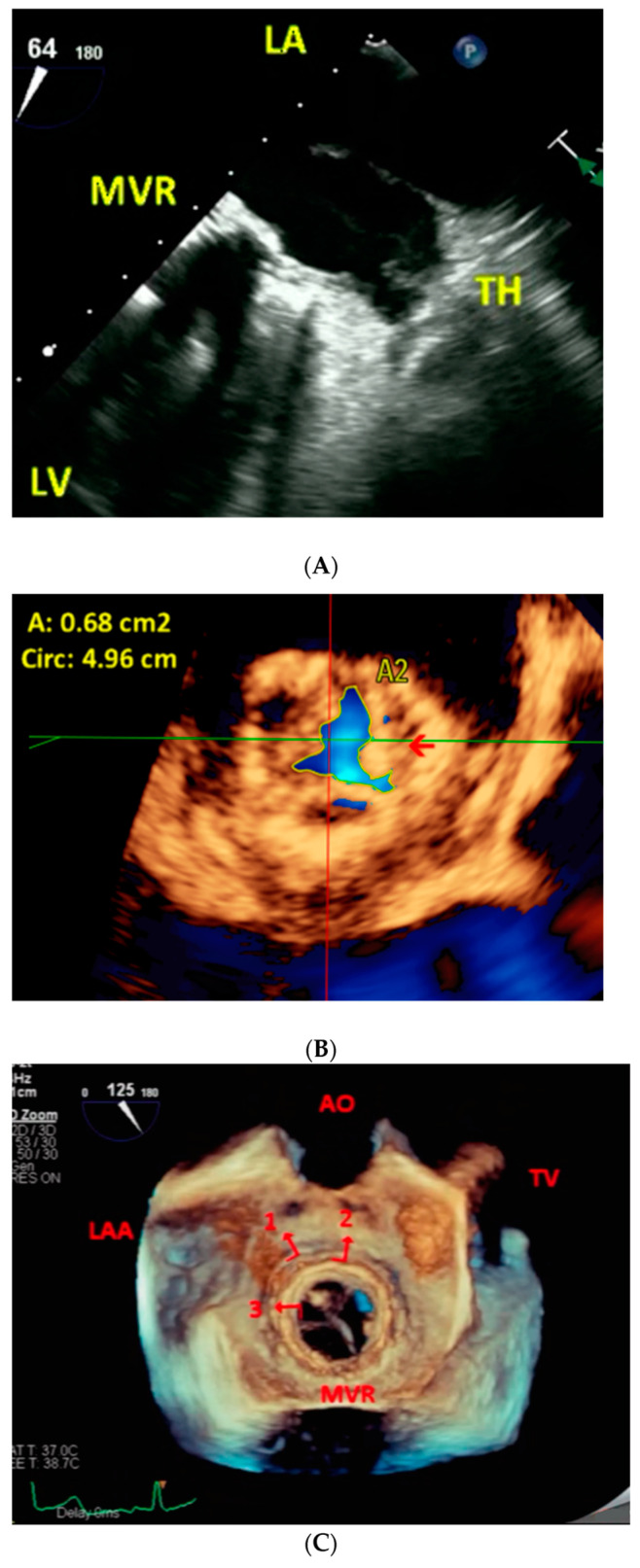
(**A**–**C**). The same patient (Case 2 above) returned 2 years and 8 months later with severe stenosis of the new MVR. Pre-procedure. Two-dimensional transesophageal echocardiography (**A**). A much smaller and poorly organized residual confined to LAA is noted in A. Live/real-time three-dimensional transesophageal echocardiography with color Doppler (**B**). The MVR orifice (red arrow) viewed in MPR mode in B measures 0.68 cm^2^ by direct planimetry indicative of severe restenosis. Post-procedure. Live/real-time three-dimensional transesophageal echocardiography (**C**). A short-axis view in full volume mode shows all the three MVR rings in C. # 1 points to the outermost ring of the original surgically implanted MVR. # 2 shows the middle ring of the first transcatheter implanted MVR. # 3 demonstrates the innermost ring of the second transcatheter implanted MVR. Abbreviations as in previous Figures.

**Figure 4:**
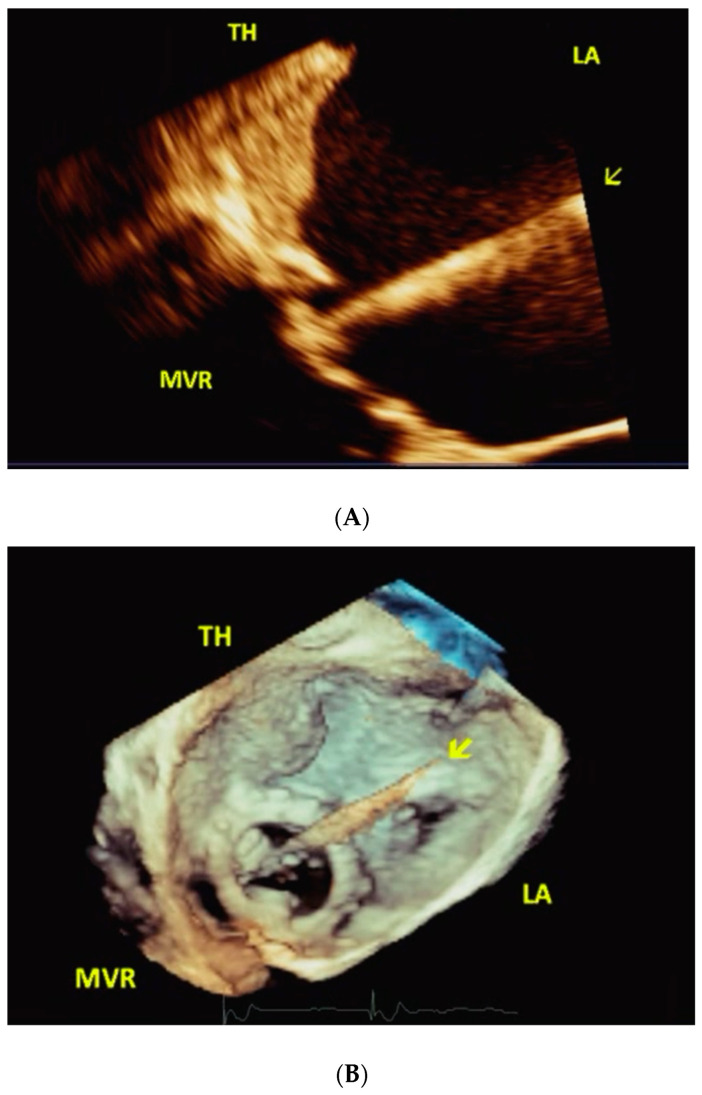
(**A**,**B**). 67-year-old male with severe MVR stenosis (Case 4). During procedure. Live/real-time three-dimensional transesophageal echocardiography (**A**,**B**). The arrow points to the catheter deployment device passing through MVR and bypassing the TH in MPR mode in A and in full volume mode in B Abbreviations as in previous Figures.

**Figure 5:**
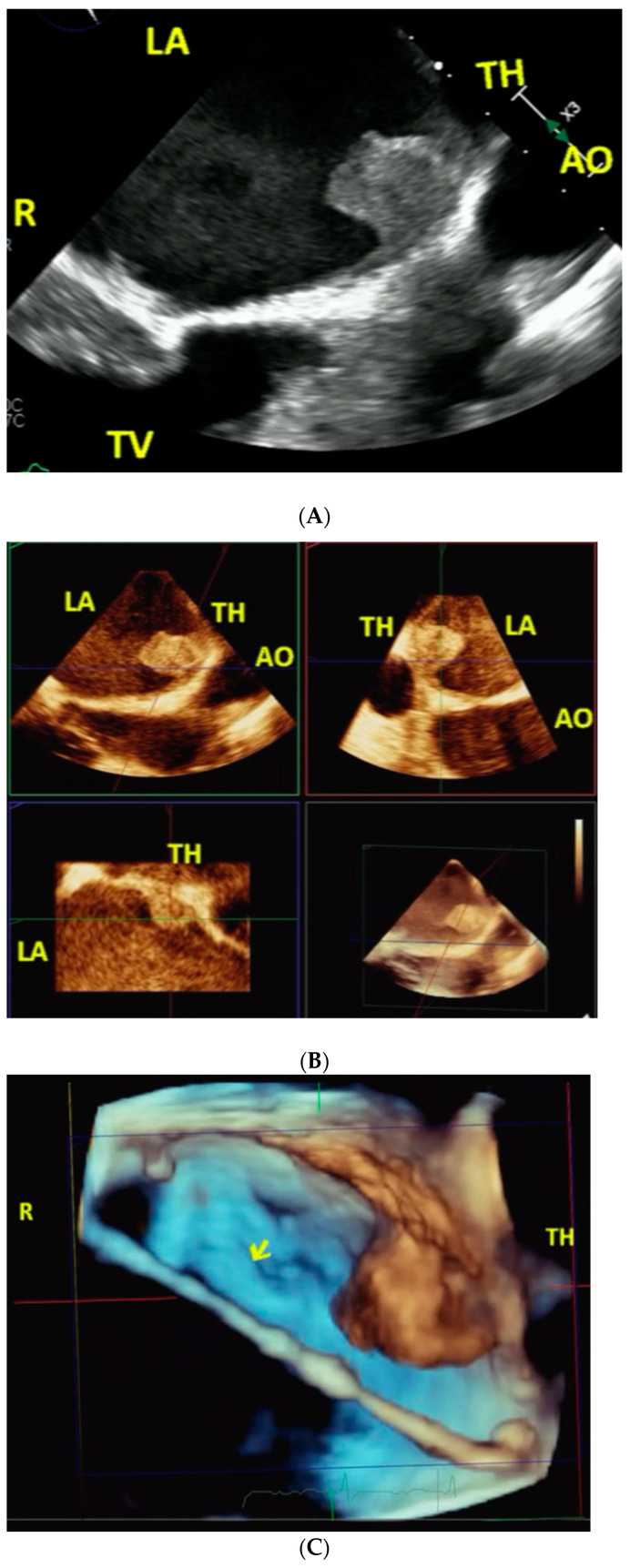

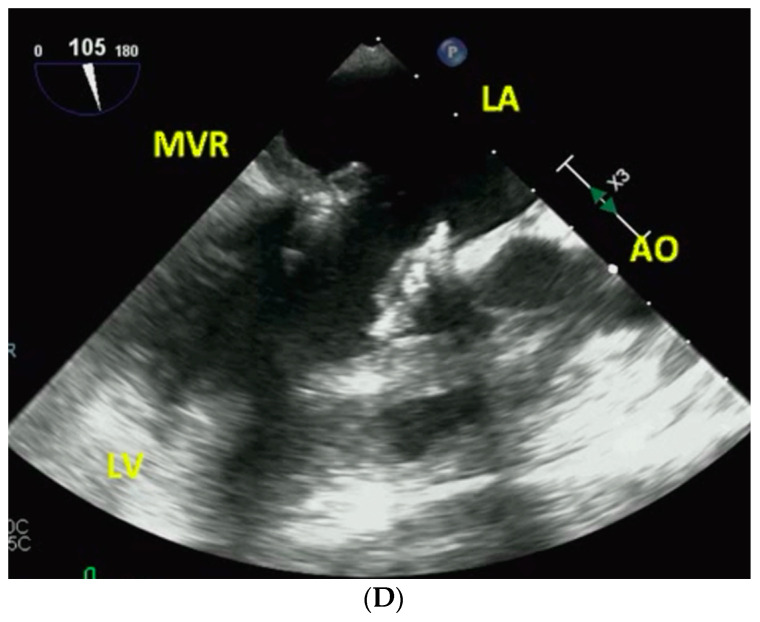
(**A**–**D**), Video S2. 58-year-old female with severe mitral valve stenosis following mitral annuloplasty ring implantation 4 years previously (Case 8). Pre-procedure. Two-dimensional transesophageal echocardiography (**A**). Shows a large TH (arrow) attached to the LA body wall adjacent to AO in A. Spontaneous contrast echoes suggestive of a low flow state are also noted adjacent to the TH. Live/real-time three-dimensional transesophageal echocardiography (**B**). The large TH attached to the LA body viewed in different cutting planes is demonstrated in B together with spontaneous contrast echoes. Areas of echolucencies noted within the thrombus are consistent with thrombus lysis. During Procedure. Live/real-time three-dimensional transesophageal echocardiography (**C**, Video S2). The arrow in C points to the catheter deployment device passing through the ring (R) and avoiding the thrombus in full volume mode (Video S2). Post-procedure. Two-dimensional transesophageal echocardiography (**D**). Demonstrates the new prosthesis in the fully open position in D. Abbreviations as in previous Figures.

During the transcatheter procedure, all patients underwent 2DTEE and 3DTEE using a Philips (Philips Ultrasound Inc, Bothell, WA, USA) EPIQ 7 system and an x7-2t or x8-2t transducer The mean pressure gradient across the degenerated bioprosthetic MV by 2DTEE ranged from 10 and 24 mmHg and the MV orifice area by planimetry using 3DTEE ranged from 0.56 to 1.09 cm^2^ in all patients with stenosis. In the 2 patients with bioprosthetic MV regurgitation, the vena contracta areas by 3DTEE were 1.25 cm^2^ and 0.44 cm^2^, respectively, indicative of severe regurgitation. Eight patients had thrombus by both 2D and 3DTEE in the LAA extending to the adjacent LA body, three patients had thrombus confined to LAA and the remaining patient had thrombus located in the LA body only. In the patient with a second procedure, the thrombus was confined to LAA. In all patients including the patient with a redo procedure, the thrombus was larger by 3DTEE as compared to 2DTEE with the thrombus area ranging from 1.39 to 17.74 cm^2^ by 3DTEE. The thrombus volume by 3DTEE calculated using the TOMTEC (TOMTEC Imaging Systems GmbH, Unterschleissheim, Germany) software ranged from 1 to 26 mL. The thrombus was noted to be mobile in 2 patients and another patient with a fixed thrombus had mobile components. These were detected by both 2D and 3DTEE. Areas of echolucencies within the thrombus consistent with thrombus lysis/dissolution were noted in all patients by both 2D and 3DTEE [8,9]. The maximum echolucent area in each thrombus was found to be larger by 3DTEE than 2DTEE (Table 2). Left and right ventricular function and other associated echocardiographic findings in each patient are also listed in Table 2. One patient with a previous successful transcatheter aortic valve replacement was noted to have developed severe prosthetic aortic valve stenosis with a mean pressure gradient of 41 mmHg.

In all patients, systematic and meticulous cropping of 3D datasets was used to comprehensively evaluate as much of the LA and LAA as possible to exclude any additional thrombus or any other structural abnormality which could pose a hazard during the transcatheter procedure. Determination of the size of the prosthetic MV to be implanted and other parameters such as left ventricular outflow tract size were determined using echocardiography and computed tomography scans as described previously [10].

## 3. Procedure and Results

The transcatheter MV implantation procedure was conducted in a standard manner under general anesthesia in all patients using the transapical approach [11]. All patients received anticoagulation with oral anticoagulants before and permanently after the procedure. The Sentinel brain protection device [12] was implanted during the procedure in only two patients. Under both 2DTTE and 3DTEE vision, a guidewire was inserted into the left ventricle through a small incision in the fifth or sixth intercostal space in the apical area guided by 2DTTE. Using both 2D and 3DTEE guidance in addition to fluoroscopy, the guidewire was advanced into the malfunctioning bioprosthesis/ring. Thereafter, it was very carefully and slowly advanced into the LA body and left upper pulmonary vein making sure there was no contact with the thrombus whose location and size were well displayed by both 2D and 3DTEE. Biplane imaging by 2DTEE and careful and systematic cropping of 3DTEE datasets were most helpful in delineating under direct vision the relationship between the thrombus and guide wires used as well as the larger prosthesis delivery system. Thus, thrombus fragmentation and/or dislodgement were avoided. All transcatheter implantations were successfully performed as previously described using a balloon-expandable transcatheter heart valve (Edward Sapien 3, XT, Edwards Lifesciences, Irvine, CA, USA or Lotus, Boston Scientific, Marlborough, MD, USA) [13]. The average duration of the procedure was 122.8 min, with a range of 54 to 210 min. The patient with associated severe prosthetic aortic valve stenosis also underwent successful redo transcatheter aortic valve in valve implantation during the same procedure. In all patients, the newly implanted prosthetic MV in valve showed unrestricted motion with no paravalvular and none or only trivial valvular regurgitation. The mean mitral prosthetic valve gradients following implantation ranged from 2 to 7 mmHg. None of the patients showed any clinical, neurological, laboratory or other evidence of embolization during or immediately following the procedure as well as during the follow up period which ranged from 3 to 36 months with a mean of 21 months. LAA/LA body thrombus did not resolve completely in any patient during the follow up period but a decrease in size was noted in all of them. Both left and right ventricular function as well as other associated valvular and other findings remained unchanged as compared to before the procedure. All patients also had amelioration of their symptoms.

## 4. Discussion

To the best of our knowledge, this is the first report describing a group of patients in whom transcatheter prosthetic MV implantation utilizing the transapical method was accomplished for a severely degenerated MV prosthesis or severe native MV stenosis status post a surgically inserted mitral annuloplasty ring with co-existing thrombus in the LAA and/or LA body. In a prior publication from our group, comparing surgical versus transcatheter MV in prosthetic MV patients, two patients with thrombus in the LA were listed in a table, but there was no further mention and no other details were provided in the published manuscript [11]. Since fluoroscopy does not visualize intracardiac thrombi, use of both 2D and 3DTEE was essential in finding the location and other attributes of the thrombi such as their size and extent in our patients and guiding the transcatheter procedure such that there was no contact with the thrombus at any time. Although in all our patients, the procedure could be successfully performed avoiding any disruption of the thrombus, it may be difficult to avoid it if the thrombus involved the prosthetic valve or was very close to it or was closely related to other anatomic landmarks normally used during the procedure such as the left upper pulmonary vein. However, in all our patients, the thrombus did not involve any of these sites by both 2D and 3DTEE further ensuring a successful outcome without any complications. More recently, transseptal rather than the apical approach has been used for transcatheter MV implantation but we do not know whether this procedure can be safely used in patients with thrombus in LAA/LA body.

3DTEE provided incremental value over 2DTEE in more comprehensively assessing both the LAA and LA body for the presence and exact sites of location of thrombus, its size, shape and mobility characteristics as well as thrombus volume which is a superior parameter of size than thrombus area measured by planimetry [9,14,15]. These findings helped the operator in carefully guiding the guide wire and the prosthesis deployment system as they traversed through the degenerated bioprosthetic MV/native stenotic MV and annuloplasty ring into the left atrium bypassing the thrombus. In all our patients, 3DTEE, unlike 2DTEE, also facilitated en face view of the degenerated bioprosthetic MV leaflets which permitted accurate measurement of the orifice area by planimetry [16]. In addition, color Doppler en face views of vena contracta of the mitral regurgitant jet facilitated measurement of vena contracta areas permitting more accurate assessment of regurgitation severity [17]. 3DTEE also provided increased confidence level during deployment of the MV prosthesis. Another additive value of 3DTEE was the ability to ensure the absence of any residual valvular or paraprosthetic MR following the procedure with a greater degree of certainty than using 2DTEE alone since the prosthesis could be viewed in multiple projections.

## 5. Conclusions

Our series of 12 patients shows the feasibility of successfully performing transcatheter bioprosthetic MV in surgically implanted degenerated MV prostheses or severe native MV stenosis following a surgically implanted mitral annuloplasty ring in patients with thrombus present in the LAA and/or LA body. All of these patients had a high or prohibitive risk for repeat operation and all absolutely refused redo surgery. Utilization of both 2D and 3DTEE especially 3DTEE was essential in avoiding contact with the thrombus and preventing any embolic complications in these exceptional cases. Our number of cases is small, and a much larger study is needed to assess the safety and efficacy of this procedure in such patients.

## Figures and Tables

**Table 1:** Patient Characteristics.

Case #. Age/Sex	Transcatheter MVR/Date/Other Interventions	Associated Comorbidities	Pre Procedure Surgical MVR/Date/Other Interventions
Case 1. 74/F	Boston Lotus, 27 mm/1/6/2016	CKD, DM, HTN, PAD, CAD, chronic AF	Tissue MVR for severe MR, TVA for severe TR/2011
Case 2. 56/F	Boston Lotus, 27 mm/1/11/2016	ESRD, chronic hepatitis B with acute exacerbation, CAD, HTN	Tissue MVR for severe MR, 31 mm/2003
Case 3. 55/M	Edwards SAPIEN, 26 mm/3/14/2016	Pulmonary hypertension, chronic AF	Tissue MVR for severe MR, TVA for severe TR/09/20/2010
Case 4. 67/M	Edwards SAPIEN XT, 29 mm and redo AVR for severe AS/11/14/2016	CAD, chronic AF	Tissue MVR for severe MR, tissue AVR for severe AR/2009
Case 2. 58/F	Edwards SAPIEN XT, 29 mm/9/21/2018	Cardiogenic shock, ESRD, HTN, CAD, chronic hepatitis B with acute exacerbation	Tissue MVR for severe MR, 31 mm/2003, transcatheter MVR for severe MS/1/11/2016
Case 5. 61/M	Edwards SAPIEN XT, 29 mm/11/4/2018	HBV carrier, chronic AF, CAD	Tissue MVR for severe MR/11/13/2009
Case 6. 64/F	Edwards SAPIEN, 26 mm/4/29/2020	COPD, antithrombin III deficiency, obstructive sleep apnea	Tissue MVR for severe MR, TVA for moderate TR/11/04/1998
Case 7. 73/M	Edwards SAPIEN, 39 mm/5/12/202	CKD, hepatocellular carcinoma, COPD, CAD, idiopathic pulmonary fibrosis, polyangiitis, chronic AF	Tissue MVR for severe MS, TVA for severe TR/05/18/2009
Case 8. 58/F	Edwards SAPIEN, 26 mm/10/27/2020	Pulmonary hypertension, chronic AF	MVA with Sorin ring for severe MS, TVA for severe TR/2016
Case 9. 68/F	Edwards SAPIEN, 29 mm/12/29/2020	DM, ESRD, HTN, CAD, severe pulmonary hypertension, liver cirrhosis	Tissue MVR for severe MR, TVA for severe TR/09/30/2011
Case 10. 78/F	Edwards SAPIEN, 26 mm/5/10/2021	CAD, chronic AF, adenocarcinoma of lung	Tissue MVR for severe MS/2011
Case 11. 67/F	Edwards SAPIEN, 29 mm/11/7/2016	Stroke, chronic AF	Tissue MVR for severe MR, TVA for severe TR/03/13/2007
Case 12. 74/F	Edwards SAPIEN XT, 29 mm/7/24/2018	Right breast cancer, hepatitis C, chronic AF	Tissue MVR for severe MR, TVA for severe TR/08/14/1996

AF = Atrial fibrillation, AS = aortic stenosis, AVR = Aortic valve replacement, CAD = Coronary artery disease, CKD = Chronic kidney disease, COPD = Chronic obstructive pulmonary disease, DM = Diabetes mellitus, ESRD = End-stage renal disease, HTN = Hypertension, MR = Mitral regurgitation, MS = Mitral stenosis, MVA = Mitral valve annuloplasty, MVR = Mitral valve replacement, PAD = Peripheral artery disease, TR = Tricuspid regurgitation, TVA = Tricuspid valve annuloplasty.

**Table 2:** Two- and three-dimensional transesophageal echocardiographic findings.

Case #. Date of MV in V	Severe MVR Stenosis/MPG (mmHg) or Severe MVR Regurgitation by 2D Echo	MVA by 3D Echo Planimetry (cm^2^)	Thrombus Location by 2D Echo and 3D Echo	Largest Thrombus Area by 2D Echo Planimetry(cm^2^)			Largest Thrombus Area by 3D Echo Planimetry (cm^2^)			Thrombus Mobility by 2D Echo and 3D Echo	Largest Echolucency in Thrombus by 2D Echo (cm^2^)	Largest Echolucency in Thrombus by 3D Echo (cm^2^)	Thrombus Volume by 3D Echo (mL)	Associated Findings
				Total	LAA	LA Body	Total	LAA	LA BODY					
Case 1. 1/6/2016	Stenosis/11	1.01	LAA + LA body	1.5	1.1	0.4	1.68	1.13	0.55	No	0.18	0.48	1.0	Mild MR, mild TR, mild AR, mild PR, normal LV/RV function
Case 2. 1/11/2016	Stenosis/19	1.09	LAA + LA body	2.0	1.2	0.8	2.68	1.86	0.82	Yes	0.07	0.53	3.7	Mild MR, moderate TR, normal LV/RV function
Case 3. 3/14/2016	Stenosis/12	1.07	LAA + LA body	2.4	2.1	0.3	2.85	2.24	0.61	Yes	0.12	0.47	2.0	Mild MR, mild TR, mild AR, normal LV/RV function
Case 4. 11/14/2016	Stenosis/12	0.84	LAA + LA body	4.7	3.0	1.7	6.38	4.44	1.94	No	0.33	0.60	6.6	Mild MR, mild TR, mild AS with peak/mean gradient 37/17.5 mmHg, normal LV/RV function
Case 2. 9/21/2018	Stenosis/17	0.68	LAA	0.85	0.85	0.0	1.39	1.39	0.0	No	0.16	0.34	3.8	Mild MR, mild to moderate TR, normal LV function, severely reduced RV function
Case 5. 11/4/2018	Stenosis/24	1.04	LAA + LA body	1.3	1.2	0.1	2.02	1.53	0.49	No	0.02	0.19	2.6	Mild MR, mild TR, trivial AR, normal LV/RV function
Case 6. 4/29/2020	Stenosis/17	0.86	LAA + LA body	2.0	1.9	0.1	4.05	3.43	0.66	No	0.0	0.30	2.8	Mild MR, mild TR, trivial AR, normal LV/RV function
Case 7. 5/12/2020	Stenosis/10	0.7	LA body	2.4	0.0	0.0	5.3	0.0	0.0	No	0.43	1.27	8.7	Mild TR, normal LV/RV function
Case 8. 10/27/2020	Stenosis/14	0.56	LA body	4.1	0.0	0.0	4.22	0.0	0.0	No	0.04	0.82	6.0	Mild MR, mild TR, normal LV/RV function
Case 9. 12/29/2020	Stenosis/15	0.76	LAA + LA body	6.3	5.9	0.4	7.77	6.92	0.85	No	1.2	2.46	14.1	Mild MR, mild TR, normal LV function, severely reduced RV function
Case 10. 5/10/2021	Stenosis/13	0.72	LAA + LA body	10.5	3.9	6.6	17.74	6.12	11.6	Yes	0.37	2.33	26	Mild MR, moderate TR, mild AR, mild PR, normal LV/RV function
Case 11. 11/7/2016	Regurgitation	*	LAA	0.96	0.96	0.0	1.7	1.7	0.0	No	0.12	0.38	1.3	Trivial MR, mild TR, normal LV/RV function
Case 12. 7/24/2018	Regurgitation	*	LAA	1.1	1.1	0.0	2.55	2.55	0.0	No	0.13	0.23	1.1	Mild TR, normal LV/RV function

2D echo = Two-dimensional transesophageal echocardiography, 3D echo = Three-dimensional transesophageal echocardiography, AR = Aortic regurgitation, AS = Aortic stenosis, LA = Left atrium body, LAA = Left atrial appendage, LV = Left ventricle, MPG = Mean pressure gradient, MR = Mitral regurgitation, MVA = Mitral valve orifice area, MV in V = Transcatheter mitral valve in bioprosthetic mitral valve procedure, MVA = Prosthetic mitral valve area, MVR = Mitral valve replacement, PR = Pulmonary regurgitation, RV = Right ventricle, TR = Tricuspid regurgitation. Largest thrombus area by 2D echo and 3D echo and largest echolucency in thrombus by 2D echo and 3D echo are in cm^2^. * MR vena contracta area by 3D planimetry in cases 11 and 12 was 0.44 cm^2^ and 1.25 cm^2^, respectively.

## Data Availability

All patient data kept confidentially with Cheng Hsin General Hospital, Taipei, Taiwan.

## Supplementary Materials

The following supporting information can be downloaded at: https://www.mdpi.com/article/10.3390/jcm11237084/s1, Video S1: Shows both fixed (left arrow) and mobile (right arrow) components of the thrombus in left atrium. Video S2: Shows catheter deployment device passing through the mitral annuloplasty ring and avoiding the thrombus in the left atrium.
